# Aggressiveness of Differentiated Thyroid Carcinoma in Pediatric Patients Younger Than 16 years: A Propensity Score-Matched Analysis

**DOI:** 10.3389/fonc.2022.872130

**Published:** 2022-04-26

**Authors:** Soo Young Kim, Hyeok Jun Yun, Hojin Chang, Seok-Mo Kim, Soyoung Jeon, Sujee Lee, Yong Sang Lee, Hang-Seok Chang, Cheong Soo Park

**Affiliations:** ^1^ Department of Surgery, Ajou University School of Medicine, Suwon, South Korea; ^2^ Department of Surgery, Thyroid Cancer Center, Institute of Refractory Thyroid Cancer, Gangnam Severance Hospital, Yonsei University College of Medicine, Seoul, South Korea; ^3^ Biostatistics Collaboration Unit, Yonsei University College of Medicine, Seoul, South Korea; ^4^ Department of Surgery, CHA Ilsan Medical Center, Goyang-si, South Korea

**Keywords:** thyroid cancer, pediatric thyroid cancer, differentiated thyroid cancer, propensity score matched analysis, papillary thyroid cancer

## Abstract

**Background:**

The biological behavior of thyroid cancer in children has been known to be different from that in adults. We sought to understand the differences between DTC presentation in pediatric (<16 years) and adult patients, to guide better prognosis and clinical treatments.

**Methods:**

This retrospective study included 48 pediatric patients younger than 16 years who underwent initial thyroid surgery and were diagnosed with DTC between January 1992 and December 2014 at Yonsei University in Seoul, South Korea. For a 1:4 propensity score-matched analysis, adult patients with matched sex and cancer size were included.

**Results:**

The mean age was 12.54 ± 3.01 years. Total thyroidectomy (70.8%) without lateral lymph node dissection (47.9%) was the most commonly performed surgery. Central (73.9%) and lateral neck node metastases (62.5%) were common; distant metastasis was observed in 2 (4.2%) patients and recurrence occurred in 11 (22.9%). In propensity score-matched analysis, central lymph node metastasis and lateral neck node metastasis were significantly more frequent in pediatric patients. Symptoms were more common in the pediatric group than in the adult group (p < 0.001). In stratified cox regression, pediatric patients were more likely to experience recurrence [HR 5.339 (1.239–23.007)]. In stratified log-rank analysis, recurrence-free survival was significantly different between the adult and pediatric groups (p = 0.0209).

**Conclusion:**

DTC in the pediatric group revealed more aggressive patterns than in the adult group with the same cancer size. Central lymph node metastasis and lateral neck node metastasis were more frequent. Stratified log-rank analysis revealed that recurrence was significantly higher in pediatric patients than in matched adult patients.

## Introduction

Among thyroid carcinomas, differentiated thyroid cancer (DTC) in childhood is one of the most common endocrine cancers in pediatric patients, accounting for 90%–95% of all pediatric thyroid carcinomas (1, 2). The most common histopathological diagnosis is papillary carcinoma, followed by follicular variant of papillary thyroid carcinoma. In 66% of the reports, multifocality has been reported (3). Since it only accounts for 1.8% of all thyroid malignancies, the treatment approaches historically have been extrapolated only from adult experiences (4, 5).

The incidence of pediatric DTC has been increasing gradually for the last few decades both in Korea and throughout the world (6–10). Its incidence is almost always higher in female patients (8). The increasing incidence has been reported to be associated with radiation exposure in children (11, 12). Many reports focused on the occurrence of papillary thyroid cancer (PTC) after the nuclear reactor accident in Chernobyl in 1986 (13–15). Treatment for a prior malignancy and a history of thyroiditis were identified as common features of pediatric thyroid cancer, suggesting thyroid cancer as a second malignancy in childhood cancer survivors (16).

The biological behavior of pediatric thyroid cancer is known to be different from that in adult patients; it has been suggested that it presents at a more advanced stage in prepuberty than in puberty (17). Younger pediatric patients are associated with a larger mean tumor size, more aggressive pathological features, and higher incidence of loco-regional and distant metastasis (18, 19). The patients first presented with symptoms such as neck swelling with a palpable mass or discomfort (3, 20).

Until now, the reported risk factors for poor prognosis are younger age, male sex, large primary tumor size, extrathyroidal tumor extension, palpable lymph nodes, distant metastases at diagnosis, and diffuse sclerosing pathology (19, 21). Despite the aggressive disease presentation and higher risk of recurrence, pediatric thyroid cancer is associated with an excellent prognosis (22–25).

Total thyroidectomy and radioactive iodine are recommended as the best management options to reduce the incidence of lung metastasis (22. One study also suggests more extensive cervical lymph node dissection with thyroid stimulating hormone (TSH) suppression therapy since young patients present with more lymph node metastasis (19). Treatment for pediatric thyroid cancer is still controversial because of these reasons.

Most previous studies on pediatric thyroid cancer also included adolescent patients; thus, we aimed to study DTC features in pediatric patients younger than 16 years.

## Material and Methods

### Study Patients

This retrospective cohort study initially included 48 pediatric patients (<16 years of age) who underwent initial thyroid surgery and were diagnosed with DTC between January 1992 to December 2014 at Yonsei University in Seoul, South Korea. Four patients were excluded from the study since they had missing information regarding pathology and clinical characteristics, i.e., a total of 48. Since the electronic chart system was introduced only in 2003, adult patients were enrolled from January 2003 to December 2014 for propensity score-matched analysis. To minimize the bias from differences in follow-up duration, for the matched analysis, data of only pediatric patients from January 2003 to December 2014 were analyzed, amounting to 37 patients in total.

### Ethics Consideration

Written informed consent by the patients was waived due to the retrospective nature of the study. The study protocol was approved by the Institutional Review Board of Yonsei University (IRB 3-2019-0281), Seoul, South Korea.

### Statistical Analysis

All statistical analyses were performed using SAS version 9.4 (SAS Institute Inc., Cary, NC, USA) and R statistics 4.0.2 (http://www.r-project.org). We conducted 1:4 matching using propensity score, selecting adult patients with the same tumor size and sex.

Continuous variables included mean and standard deviation, and categorical variables included frequency and percentage. To compare continuous and categorical variables between two groups, a generalized estimated equation was used. Recurrence-free survival curves were plotted using the Kaplan-Meier method, and the stratified log-rank test was performed to compare the recurrence rate between pediatric and adult patients. To evaluate risk factors for recurrence, stratified Cox regression was performed and are presented as hazard ratio (HR) and 95% confidence interval (CI). P-values of <0.05 were deemed to indicate statistical significance.

## Results

The baseline clinical characteristics of the 48 included patients are shown in **Table 1**. The incidence of DTC was higher in female patients (77.1%), and the mean age was 12.54 ± 3.01 years. The most common presenting symptom was a neck mass (60.4%). Most of the patients (85.4%) did not have a family history of thyroid cancer. Total thyroidectomy (70.8%) without lateral lymph node dissection (47.9%) was the most commonly performed surgery. Papillary thyroid cancer (87.5%) followed by follicular cancer (12.5%) were the most common diagnoses. Central (73.9%) and lateral neck node metastases (62.5%) were common. Further, distant metastasis was observed in 2 (4.2%) patients and recurrence occurred in 11 (22.9%) patients.

**Table 1:** The baseline clinical characteristics of 48 pediatric patients.

Clinicopathologic features		N=48 (%)
**Age (years)**		12.54 ± 3.01
**Sex**	**Male**	11 (22.9)
	**Female**	37 (77.1)
**Tumor size (cm)**		2.42 ± 1.45
**Presenting symptom**	**None**	18 (37.5)
	**Mass**	29 (60.4)
**Family History**	**No**	41 (85.4)
	**Thyroid cancer**	4 (8.3)
	**Non-thyroid cancer**	3 (6.3)
**OP**	**Less than total**	14 (29.1)
	**Total**	34 (70.8)
**LND**	**None**	23 (47.9)
	**Unilateral**	14 (29.2)
	**Ubilateral**	6 (12.5)
	**Selective**	2 (4.2)
	**mediastinal**	3 (6.3)
**Histology**	**Papillary**	42 (87.5)
	Conventional	29/42 (69.0)
	Diffuse sclerosing variant	10/42 (23.8)
	Not otherwise specified	3/10 (7.2)
	**Follicular**	6 (12.5)
**Central node metastasis**		34 (73.9)
**Lateral neck node metastasis**		30 (62.5)
**Distant metastasis**		2 (4.2)
**RAI therapy**		31 (64.6)
**Recurrence**		11 (22.9)
**F/u months**		158 ± 83

We performed 1:4 propensity score matching with adult patients with matched sex and tumor size; the results are shown in **Table 2**. Pediatric patients were more likely to be symptomatic than adult patients (p < 0.001). There were no differences regarding family history and surgical extent. Histology was not significantly different when distinguished into papillary and follicular cancer. However, further dividing into histological variants of papillary thyroid cancer showed significant difference. Diffuse sclerosing variant of papillary thyroid carcinoma was more frequent in pediatric thyroid cancer [9/34 (26.5%) vs 6/145 (4.1%)]. Central lymph node metastasis [27 (73.0%) vs 69 (46.6%)] and lateral neck node metastasis were significantly more frequent in pediatric patients than in matched adult patients. There were no significant differences in distant metastasis and use of RAI therapy. Recurrence was significantly more frequent in pediatric patients than in adult patients (13.5% vs 2%, p = 0.0131).

**Table 2:** 1:4 Propensity score-matched GEE analysis.

		Pediatric (N=37)	Adult (N=148)	p-value
**Age**		12.76 ± 2.65	45.67 ± 13.19	<0.001
**Sex**	Male	7 (18.9)	31 (20.95)	>0.9999
	female	30 (81.1)	117 (79.05)	>0.9999
**Size (cm)**		2.22 ± 1.43	2.23 ± 1.43	>0.9999
**Presenting symptom**				<0.001
	None	17 (45.9)	120 (81.1)	
	Mass	19 (51.4)	25 (16.9)	
	Hoarseness	0	3 (2.0)	
	Other	1 (2.7)	0 (0.0)	
**Family history**				0.402
	No	30 (81.1)	134 (90.5)	
	Thyroid cancer	4 (10.8)	7 (4.7)	
	Non-thyroid cancer	3 (8.1)	7 (4.7)	
**OP**				0.561
	Less than total	9 (24.3)	30 (20.3)	
	Total	28 (75.7)	118 (79.7)	
**LND**				<0.001
	None	18 (48.7)	111 (75.1)	
	Unilateral	8 (21.6)	27 (18.2)	
	Bilateral	6 (16.2)	7 (4.7)	
	selective	2 (5.4)	0	
	mediastinal	3 (8.1)	3 (2.0)	
**Histology**				0.072
	**Papillary**	34 (91.9)	145 (98.0)	
	Conventional	24/34 (70.6)	111/145 (76.6)	<0.001
	Diffuse sclerosing variant	9/34 (26.5)	6/145 (4.1)	
	Follicular variant	0	22/145 (15.2)	
	others	0	6/145 (4.1)	
	Not otherwise specified	1/34 (2.9)	0/145	
	**Follicular**	3 (8.1)	3 (2.0)	
**Multiplicity**				0.836
	No	25 (67.6)	102 (68.9)	
	Unilateral	4 (10.8)	11 (7.4)	
	Bilateral	8 (21.62)	35 (23.7)	
**T stage**				0.317
** T1**		10 (27.0)	35 (23.6)	
** T2**		2 (5.4)	21 (14.2)	
** T3**		24 (64.9)	81 (54.7)	
** T4**		1 (2.7)	11 (7.4)	
**Central node metastasis (N1a)**		27 (73.0))	69 (46.6)	0.0008
**Lateral neck node metastasis (N1b)**		24 (64.9)	37 (25.0)	<0.001
**Distant metastasis (M1)**		1 (2.7)	3 (2.0)	0.817
**RAI therapy**		25 (67.6)	114 (77.0)	0.210
**Recurrence**		5 (13.5)	3 (2.0)	0.0131
**Time to recur (days)**		1323 ± 1384	678 ± 182	0.2497
**F/u months (days)**		3688 ± 1281	2695 ± 1143	<0.001

In univariable stratified cox regression analysis, pediatric patients were more likely to experience recurrence. Pediatric patients had approximately 5.4 times the risk of recurrence than adult patients [HR 5.339 (1.239–23.007)]. Multiplicity of disease was associated with recurrence compared with a single disease [HR 264.171 (3.219–21678.05)]. In multivariable analysis, only multiplicity was associated with recurrence (**Table 3**).

**Table 3:** Stratified Cox regression to evaluate risk factors for recurrence.

Variable	Univariable model		Multivariable model	
	HR (95%CI)	p-value	HR (95%CI)	p-value
**Pediatric patient (age 0-15)**				
No	Ref			
Yes	5.339 (1.239-23.007)	0.0246	2.937 (0.233-36.974)	0.4045
**OP name**				
Less than total	Ref			
Total	6.213 (0.193-200.173)	0.3026		
**Histology**				
Papillary	Ref			
Follicular	0.474 (0.005-46.611)	0.7498		
**Central lymph node metastasis**				
No	Ref			
Yes	13.094 (0.565-303.675)	0.1088		
**Lateral lymph node metastasis**				
No	Ref			
Yes	17.617 (0.801-387.621)	0.0689		
**Multiplicity**				
No	ref			
Unilateral	264.171 (3.219-21678.05)	0.0131	55.092 (1.640-1850.663)	0.0254
Bilateral	18.946 (0.780-460.079)	0.0707	6.712 (0.491-91.702)	0.1535

**Figure 1:**
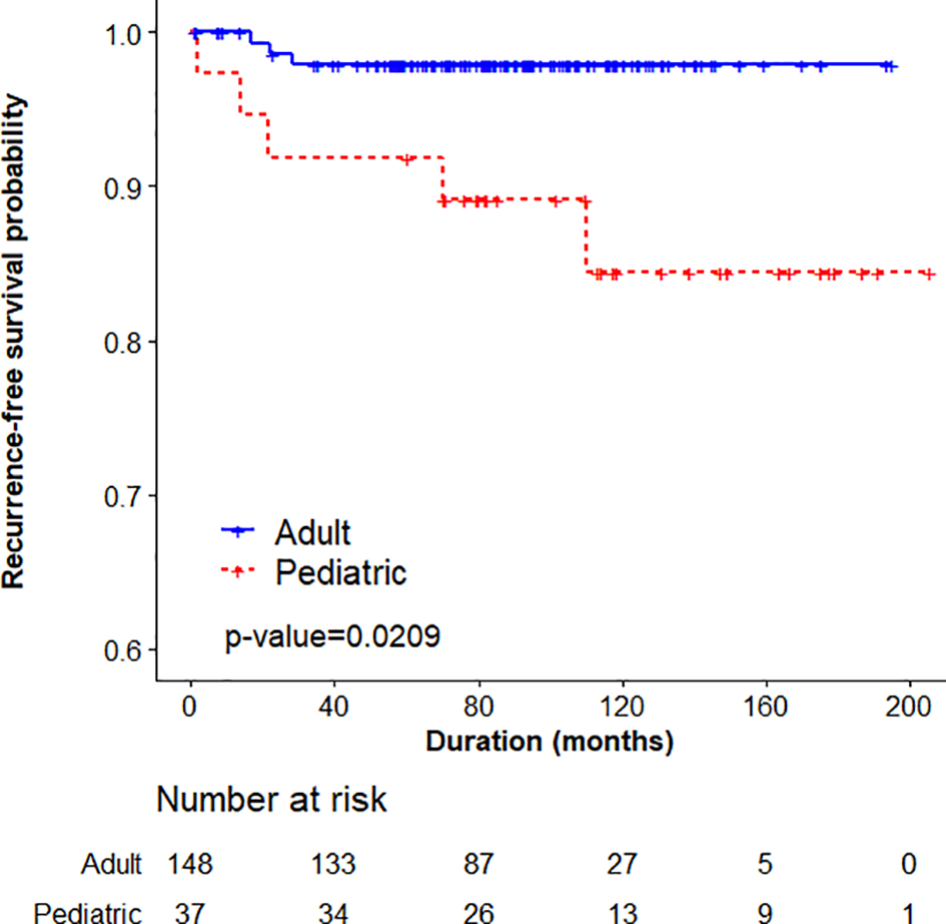
Stratified log-rank analysis comparing adult and pediatric patients.

In stratified log-rank analysis, recurrence-free survival was significantly different between the adult and pediatric groups (p = 0.0209; **Figure 1**).

## Discussion

To the best of our knowledge, this is the first study to examine such a large number of young pediatric patients under the age of 16 years with propensity score matching. Previous studies also included adolescent patients and did not consist of pediatric patients solely (8, 16, 20, 26, 27).

Pediatric DTC is an uncommon malignancy. Although the disease is more aggressive and presents at more advanced stages in this group, the overall prognosis is known to be excellent. Our results show that pediatric patients younger than 16 years have a higher risk of recurrence than adult patients with the same tumor size and matched sex. Pediatric patients showed significantly more central node metastasis and lateral neck node metastasis, which could have had an impact on the higher recurrence probability.

Pediatric patients were reported to have larger lesions than adult patients (median 23.6 vs 19.3 mm), with more frequent lymph node metastases (67.8% vs 42.1%) and distant metastases (19, 28). In this study, pediatric patients were matched to adult patients with the same tumor size to overcome this difference. Young patients were significantly more likely to undergo second treatment such as surgery and radiotherapy. The rate of mutations in the proto-oncogene BRAF was significantly higher in adult patients than that in pediatric patients with PTC (28).

Sherman et al. (29) noted the significance of tumor size; the small size of the thyroid gland in children can lead to earlier extrathyroidal spread of the disease. However, extrathyroidal extension was not more frequent in our study. Reports have shown that although DTC presented more aggressively at the time of diagnosis in children, intensive management elicits a similar clinical outcome in both children and young adults (18, 22). A study comparing patients younger than 10 years, and pediatric patients older than 10 and younger than 18 years, noted no significant differences in tumor size or aggressiveness; they suggested that younger children are more likely to have lymph node metastasis at presentation as well as subsequent metastases (16, 25). Our study showed that pediatric patients showed more aggressive patterns than adult patients with the same cancer size with more frequent central node and lateral node metastasis. More than half of the pediatric patients presented with cervical lymph node metastasis.

Lee et al. showed that the younger the patient at diagnosis, the higher the percentage of PTC, multifocality, ETE, LN metastasis, and lung metastasis. The age at diagnosis was not a predictor of recurrence-free survival, suggesting that multifocality rather than age at diagnosis is an important predictor for recurrence (20). Chaukar et al. suggested a correlation between hormonal influence during puberty and an increased risk of thyroid carcinoma. They observed a near equal distribution of female and male patients in the prepubertal age group (1.5:1), whereas the ratio in patients of 13 to 17 years of age was 3:1, which could suggest a hormonal influence. Since thyroid carcinoma is known to be TSH-dependent, any condition leading to an increase in circulating TSH level in the blood may be associated with an increased risk of thyroid cancer (22). Another study showed that female patients predominantly presented with DTC, although the ratio in the young group was higher, suggesting an association with sex hormone factors (19).

Race was identified as an independent predictor in multivariate analysis, suggesting that non-Caucasian patients are at higher risk of recurrence than Caucasians. Pediatric PTC was associated with lateral node involvement (30). This could somehow explain the higher incidence of recurrent pediatric cancer in our study since our study population only comprised Korean patients, and pediatric patients showed frequent lateral lymph node involvement.

In our study, central lymph node metastasis and lateral neck node metastasis were more frequent. An aggressive operative approach to lymph node resection in experienced hands may be safer when considering complication and recurrence rates compared with less complete resection at a lower volume center (30).

In young children, more stable and progressive disease forms were noted, showing higher cure rates in adolescents (19). Disease-free survival was significantly shorter in the children group than that in the adolescent group, but no significant difference was found in cancer-specific survival between these two groups (25). A previous study on the initial and dynamic risk stratification of pediatric patients showed that reflecting the extent of disease by tumor size, localized invasion, and the number and size of cervical LN metastases was useful for predicting the risk of structurally persistent or recurrent disease (31). Using the Kaplan-Meier analysis, we observed that the incidence of recurrence was significantly higher in the pediatric group than in the matched adult group.

According to the ATA management guidelines for children, total thyroidectomy remains the treatment of choice for pediatric patients with thyroid cancer (32). A study reported that comparable surgical outcome was seen with lobectomy instead of total thyroidectomy in patients with limited disease, such as those with tumors smaller than 2 cm, no lymph node metastasis, and no multifocal disease (27). Considering our results wherein the mean tumor size was 2.33 ± 1.39 cm and the fact that the central node metastasis (73.9%) and lateral neck node metastasis (64.6%) rates were quite high, patients suitable for lobectomy may be rare. Our results suggest that young patients should undergo more thorough preoperative analysis to decide the extent of surgery since loco-regional and distant metastasis may be more frequent.

This study has several limitations, including small sample size, single institution and limitations inherent to retrospective nature of the study. Molecular analysis should be included in future studies.

## Conclusion

Pediatric patients showed more aggressive patterns than adult patients with the same cancer size. Central lymph node metastasis and lateral neck node metastasis were more frequent. Recurrence was more significantly observed in stratified log-rank analysis in pediatric patients than in matched adult patients.

There is a need for prospective, collaborative multicenter studies. Future prospective multicenter pediatric studies are required to answer questions regarding the natural history of this condition in pediatric patients.

## Data Availability Statement

The data analyzed in this study is subject to the following licenses/restrictions: personal data. Requests to access these datasets should be directed to kimsuy@aumc.ac.kr.

## Ethics Statement

The studies involving human participants were reviewed and approved by Institutional Review Board of Yonsei University (IRB 3-2019-0281), Seoul, South Korea. Written informed consent from the participants' legal guardian/next of kin was not required to participate in this study in accordance with the national legislation and the institutional requirements.

## Author Contributions

SYK, YSL, SMK, HSC, CSP contributed to the conception and design of the study. SYK, HJY and HC organized the database. SL and SJ performed the statistical analyses. SYK wrote the first draft of the manuscript. All authors contributed to manuscript revision, read, and approved the submitted version.

## Funding

This research was supported by faculty research fund of Yonsei University College of Medicine (6-2019-0092) and by Basic Science Research Program through the National Research Foundation of Korea (NRF) funded by the Ministry of Science and ICT (2017R1E1A1A03070345).

## Conflict of Interest

The authors declare that the research was conducted in the absence of any commercial or financial relationships that could be construed as a potential conflict of interest.

## Publisher’s Note

All claims expressed in this article are solely those of the authors and do not necessarily represent those of their affiliated organizations, or those of the publisher, the editors and the reviewers. Any product that may be evaluated in this article, or claim that may be made by its manufacturer, is not guaranteed or endorsed by the publisher.
